# Molecular and Structural Analysis of Specific Mutations from Saudi Isolates of SARS-CoV-2 RNA-Dependent RNA Polymerase and their Implications on Protein Structure and Drug–Protein Binding

**DOI:** 10.3390/molecules27196475

**Published:** 2022-10-01

**Authors:** Mubarak A. Alamri, Muhammad Tahir ul Qamar, Alhumaidi B. Alabbas, Safar M. Alqahtani, Manal A. Alossaimi, Sikandar Azam, Muhammad Harris Hashmi, Muhammad Shahid Riaz Rajoka

**Affiliations:** 1 Department of Pharmaceutical Chemistry, College of Pharmacy, Prince Sattam Bin Abdulaziz University, Al-Kharj 11942, Saudi Arabia; 2 Integrative Omics and Molecular Modeling Laboratory, Department of Bioinformatics and Biotechnology, Government College University Faisalabad, Faisalabad 38000, Pakistan; 3 Department of Virology, Graduate School of Medicine, Tohoku University, Sendai 980-8577, Japan; 4 University of Alabama at Birmingham, Birmingham, AL 35294, USA; 5 Department of Microbial Pathogenesis, University of Maryland, Baltimore, MD 21201, USA

**Keywords:** SARS-CoV-2, COVID-19, mutations, RdRp, molecular dynamics simulation

## Abstract

The COVID-19 pandemic caused by severe acute respiratory syndrome coronavirus-2 (SARS-CoV-2) has stressed the global health system to a significant level, which has not only resulted in high morbidity and mortality but also poses a threat for future pandemics. This situation warrants efforts to develop novel therapeutics to manage SARS-CoV-2 in specific and other emerging viruses in general. This study focuses on SARS-CoV2 RNA-dependent RNA polymerase (RdRp) mutations collected from Saudi Arabia and their impact on protein structure and function. The Saudi SARS-CoV-2 RdRp sequences were compared with the reference Wuhan, China RdRp using a variety of computational and biophysics-based approaches. The results revealed that three mutations—A97V, P323I and Y606C—may affect protein stability, and hence the relationship of protein structure to function. The apo wild RdRp is more dynamically stable with compact secondary structure elements compared to the mutants. Further, the wild type showed stable conformational dynamics and interaction network to remdesivir. The net binding energy of wild-type RdRp with remdesivir is -50.76 kcal/mol, which is more stable than the mutants. The findings of the current study might deliver useful information regarding therapeutic development against the mutant RdRp, which may further furnish our understanding of SARS-CoV-2 biology.

## 1. Introduction

COVID-19 is a major pandemic of the 21st century and is caused by severe acute respiratory syndrome coronavirus-2 (SARS-CoV-2) [1,2]. The virus poses a real threat to the global health care system and has resulted in millions of deaths. As of 14:49 CEST on 14 September 2022, WHO had received reports of 607,083,820 confirmed cases of COVID-19, including 6,496,721 deaths. COVID-19 symptoms vary, but mild cases frequently experience fatigue, cough, and fever. Moderate cases may experience mild pneumonia or breathing difficulties, while severe cases may experience organ failure, severe pneumonia and death [3]. Although the COVID-19 disease is managed in many countries around the world, hundreds of cases are still reported every day [4]. Evidence suggests that SARS-CoV-2 mortality can vary significantly depending on geographic location. Differences in viral infection rates can be attributed to a variety of factors, including different national policies governing people’s movement restrictions, isolation and quarantine, as well as differences in genetic population herd immunity [5]. Therefore, substantial efforts are needed to better understand the virus’s biology to obtain useful insights for the discovery and development of novel therapeutics.

Different variants of the virus were reported in late 2020, which enabled the virus to spread quickly in the population and made it more virulent compared to the one reported in Wuhan, China [6,7]. The World Health Organization classified these variants as variants of concern and are as follows. The alpha variant (linage B.1.1.7), which emerged in the United Kingdom in the year 2020 [8]. Notable mutations in the alpha lineage are N501Y and P681H [9]. Initial analysis based on matched case-control studies in the United Kingdom revealed that the alpha variant was not significantly associated with an elevated risk of hospitalization or mortality among infected individuals. However, further investigation revealed that the variant is linked to an increase in severity, and as a result, a 61% increase in mortality rate [10]. The beta variant (lineage B.1.351) was reported from South Africa in the month of May 2020 [11]. The gamma variant was discovered in Brazil and was revealed to be more virulent and associated with transmissibility [12]. According to reports, the Gamma variant may be more severe, with a greater number of younger people presenting with advanced disease and succumbing to the virus. The delta variant (B.1.617.2), a dominant variant with eight S protein changes [13], which emerged in India, was found responsible for killing thousands of people, as the variant was seen with more transmissibility and changed antigenicity [14]. The omicron variant emerged in Botswana in 2021. Several other variants have been witnessed by WHO and are predicted to continue this cycle of genetic changes [15]. Therefore, constant efforts are required to monitor these genetic changes so they can be better controlled.

Vaccines provide protection without the risk of infection and subsequent severe symptoms, and they continue to be the most effective strategy for reducing disease burden and acquiring immune protection against SARS-CoV-2. Updating or developing new COVID-19 vaccines may thus be required to control viral variants. Current mRNA, protein and viral vaccines could be updated by replacing older S protein variants with emerging variants [10]. It is possible that both old and new forms of the S protein could be included in a single vaccine, referred to as a multivalent vaccine [16]. Although vaccinated people are at risk of reinfection, their transmission rates are lower than those of unvaccinated people. Vaccinated people also have lower disease severity, are more likely to recover and require less hospitalization. Some countries have administered booster shots to fully vaccinated people, and they have been shown to reduce severe COVID-19 and SARS-CoV-2 infections [17].

SARS-CoV-2 is an RNA virus, the genome of which is about 29.8 Kb in length [18,19]. The virus genome comprises 14 open reading frames (ORFs) and codes for 29 proteins [20]. The first two ORFs (1a and 1ab) comprise two-thirds of the genome and encode polyproteins that are proteolytically cleaved into 16 nonstructural proteins (NSP1–NSP16), including the SARS-CoV2 RNA-dependent RNA polymerase (RdRp; nsp12). The ORF1a encodes for polyprotein 1a (pp1a), which is cleaved into 11 nonstructural proteins (nsp1–nsp11), whereas ORF1b produces nonstructural proteins nsp12–16. The last third of the genome encodes other proteins, including Envelope (E), Membrane (M), Nucleocapsid (N) and Spike (S) protein [21]. The SARS-CoV2 RNA-dependent RNA polymerase (RdRp) from the structural perspective contains multiple domains and catalyzes RdRp synthesis [22]. Due to its vital functionality in the virus’s biology and survival plus the absence of its homology sequence in the host, it is an ideal target for the design and development of antiviral drugs [23]. As such, this enzyme has been successfully targeted by the remdesivir drug, which interferes with the enzyme’s functionality and is capable of inhibiting the virus [24]. In response to the drug action, the virus mutates the enzyme sequence, which results in decreased binding, making the virus escape from the drug action. 

Genomic surveillance is emerging as a vital necessity to achieve effective mitigation and containment. Since SARS-CoV-2 variants have already been identified, it is critical to obtain reliable evidence about whether they are more virulent, contagious or resistant to COVID-19 vaccines before they spread around the world. Genomic surveillance increases the availability of whole-genome data, advances phylogenetic methods and makes use of next-generation sequencing applications. These methods provide novel ways to detect variants that differ phenotypically or antigenically. Genomic surveillance allows for earlier detection and implementation of effective strategies to mitigate and contain SARS-CoV-2 variants and other novel virus outbreaks [25,26].

In this study, we evaluated several mutations of the RdRp enzyme extracted from Saudi Arabia SARS-CoV-2 isolates and aligned them with the Wuhan RdRp enzyme to highlight key mutations that effect the enzyme structure, function and dynamics and overall virus pathogenicity, transmissibility and disease mortality. The findings of this study were found to be consistent with those of Pachetti et al. [5] and Yashvardhini et al. [27], who described the RdRp of SARS-CoV-2 acquiring drug-resistance properties due to the occurrence of a high frequency of mutations in the RdRp of infected populations worldwide and in India, respectively. The findings will not only give key findings to the society of the virus pathogenic strains circulating in Saudi Arabia but also will guide the discovery of novel drugs.

## 2. Materials and Methods

### 2.1. Sequence Retrieval from NCBI Virus Database

The NCBI Virus database (https://www.ncbi.nlm.nih.gov/labs/virus/vssi/#/find-data/virus) of NCBI stores sequence and proteomic data of viruses, which can be easily accessed and can be used in biological research. The study was commenced by retrieving all RNA-dependent RNA polymerase isolated from Saudi Arabia SARS-CoV-2. The Wuhan SARS-CoV-2 RdRp was considered as reference for comparative analysis. 

### 2.2. Multiple Sequence Alignment by Clustal Omega Program

Multiple sequence alignment was performed for identification of mutations in the SARS-CoV-2 RNA-dependent RNA polymerase protein. For comparative analysis, the RNA-dependent RNA polymerase sequence from the first sequenced Chinese genome (YP_009724397) was used as a reference. Clustal Omega was used for alignment of the sequences [28]. In order to measure the impact of mutation on the enzyme structure, dynamics and stability, the free-energy difference as well as vibrational entropy energy were estimated. The mutational impact study was conducted using DynaMut program [29]. During this analysis, the crystal structure of polymerase with pdb ID of 6VYO was employed for RBD (receptor-binding domain) modeling. RBD is a virus immunogenic fragment that binds to a specific endogenous receptor sequence to gain entry into host cells, whereas RCSB protein ID: 6WJI was considered for domain dimerization analysis of N-protein. The DynaMut software guides visual understanding of fluctuations in protein structure due to mutations.

### 2.3. Secondary Structure Predictions

The secondary structure analysis was performed using an online CFSSP software [30]. CFSSP employ Chou–Fasman algorithm for predicting secondary structure elements of given amino sequence, and the output is in the form of alpha helix, beta sheet and turns. 

### 2.4. Structural Homology Modeling

The structure modeling of the mutants was performed using SwissModel [31]. Selection of the template for the modeled structure was carried out based on sequence identity score and query coverage. The Qualitative Model Energy Analysis score (QMEANDisCo Global) for models were 0.88, 0.89 and 0.89 for A97V, P323I and Y606C structures.

### 2.5. Molecular Docking Studies 

Molecular docking was performed using Autodock Vina software [32]. The X-ray structure of RNA-dependent-RNA polymerase (RdRp) with 2.50 Å resolution (BDP ID: 7BV2) [24], as well as the three models of RdRp structures with mutations were used. The protein structures were optimized by removing unwanted cocrystallized water molecules and further saved in PDB format utilizing BIOVIA Discovery Studio Visualizer 2019. The structure of remdesivir was obtained from PubChem (CID: 121304016) in PDB format. The polar hydrogen within the protein and ligand were added by Autodock Tools [33] and then all structures were saved in Autodock PDBQT format. The Autogrid tool was used for the measuring the docking grid box with 1.00 Å spacing and box dimensions of 24 Å × 22 Å × 28 Å (x, y and z) and center of 113.78 Å × 118.474 Å × 132.008 Å (x, y and z). The binding modes and interactions of remdesivir with RdRp binding site were analyzed and visualized using PyMOL [34] and UCSF Chimera 1.14 [35].

### 2.6. Molecular Dynamics (MD) Simulation

MD simulations were conducted for a production run of 20 ns for wild enzyme and mutants using AMBER20 [36]. Preprocessing of the systems involved the application of AMBER antechamber program [37]. Topology and parameter files were built using the ff14SB force field [38] and recorded through the leap module. Both systems were neutralized by adding sodium ions and placed in 12 Å TIP3P water box. Energy of the systems was minimized by running 1500 steps of the steepest descent method and 1000 rounds of the conjugate gradient method. For nonbounded interactions, the threshold distance was set to 8 Å. The systems were then heated for 50 ps at constant volume and temperature (300 K). Systems equilibration was achieved for 100 ps where periodic boundary conditions along with constant pressure and Langevin thermostat were applied [39]. Lastly, both systems were subjected to a production run of 20 ns in an explicit solvent model using the isothermal–isobaric ensemble where temperature and pressure were set to 300 K and 1 atm, respectively. To model the long-range electrostatic effects, periodic boundary conditions and particle-mesh Ewald method were used along with a coupling algorithm to couple temperature with an external bath. SHAKE algorithm [40] was employed to apply hydrogen bond constraints whereas constant temperature was achieved through the Langevin coupling integration algorithm. A time step of 2 ps was used to Newton’s equations for complex dynamics and trajectory files were created after 1 ps. The PTRAJ module [41] of AMBER was used to analyze MD trajectories.

### 2.7. Binding Free-Energy Calculations Using MMGBSA

AMBER20 MMPBSA.py module was used to calculate the binding free energy of the simulated complexes [42]. This tool estimated different components of the net binding free energy significant in interactions between the ligand and the biomolecule target. MM/GBSA is now a commonly employed technique in drug discovery practices to evaluate the affinity of ligand molecules to a specific biological target and underline key chemical intermolecular interaction energies. Estimation of the MM/GBSA binding free energy can be concluded as:

ΔGbind = ΔGcomplex − (ΔGprotein − ΔGligand)

ΔGbind = ΔEMM+ ΔEpolar + ΔESASA − TΔS

ΔEMM = ΔEelect + ΔEvdw

ΔESASA = γSASA

In the above equations, ΔG_bind_ represents the net free binding energy, -*T*Δ*S* corresponds to entropic energy and ΔE_MM_ indicates molecular mechanics potential energy and can be split into van der Waals (ΔE_vdw_) energy and electrostatic (ΔE_elect_) energy. ΔE_polar_ and ΔE_SASA_ are used for polar contributions and nonpolar solvation energy, respectively. The latter is calculated using a solvent-accessible surface area (SASA) nonpolar model. 

## 3. Results and Discussion

All of the valid submitted Saudi Arabia SARS-CoV-2 RdRp enzyme sequences found in the viral database of NCBI were retrieved (21 May 2022) for comparative sequence analysis to highlight mutations that may alter the enzyme structure and function and may contribute to the virus’s overall survival in terms of transmissibility, infectious potential and escaping from the currently available therapeutics. The accession number of the available Saudi Arabia SARS-CoV-2 RdRp enzymes is listed in Table 1. For alignment purposes, Clustal Omega was employed to perform multiple sequence alignment of the sequence whereas RdRp from Wuhan SARS-CoV-2 was used as a reference (accession number: YP_009724389). The MSA analysis identified three mutants of the RdRp in Saudi Arabia RdRp sequences. These mutants are: A97V, P323I and Y606C. A97V and Y606C mutations were observed in a single sample, while P323I was seen in 56 samples, as shown in Table 2.

### 3.1. Identification of Mutations in Structural Proteins Present in Saudi Isolates

Enzymes as proteins in nature performed their function in dynamics, which is significant for functionality and protein–protein interactions as well as for protein–ligand interactions. The impact of the identified mutations on the RdRp enzyme structure stability and functionality was determined by the mean of the free-energy differences (ΔΔG) between the Wuhan RdRp and mutants. The study of this free-energy difference can highlight the impact of mutation on the secondary structure’s stability and tertiary structure’s dynamics. In the process, vibrational entropy was estimated between the wild-type RdRp and mutants. The entropy energy anticipated the stability changes in kcal/mol, as when the ΔΔG is ≥0 it demonstrates a stabilizing effect of the mutation on the protein stability, while when ΔΔG is ≤0 it is an indication of the mutation’s destabilizing effect on the enzyme structure and overall functionality. The vibrational entropy energy (termed as ΔΔSVibENCoM) between the mutants and wild type was assessed by the ENCoM tool. The ΔΔSVibENCoM of ≥0 conferred flexibility to the RdRp enzyme, whereas ΔΔSVibENCoM ≤ 0 illustrates rigidity to the enzyme due to mutation. The free-energy difference among the mutants and between the mutants and wild type is tabulated in Table 3. The A97V and P323I were found to have a stabilizing impact on the RdRp enzyme structure and functionality, while and Y606C revealed a destabilizing impact.

### 3.2. Δ Vibrational Entropy Energy between Wild-Type and Mutant

**Figure 1:**
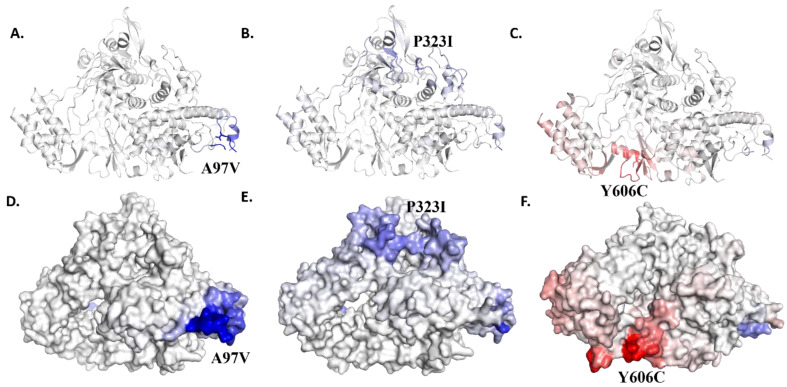
Δ Vibrational entropy energy between wild-type and mutant RdRp. Ribbon representation showing the change in vibrational entropy energy between wild-type and mutant RdRp due to (**A**) A97V and (**B**) P323I (**C**) Y606C. Surface representation showing the change in vibrational entropy energy between wild-type and mutant PLpro due to (**D**) A97V and (**E**) P323I (**F**) Y606C. Amino acids are colored according to the vibrational entropy change as a consequence of mutation of PLpro protein. Blue represents a rigidification of the structure and red represents a gain in flexibility.

The vibrational entropy energy was determined further to find out whether the identified mutant(s) can either increase or decrease flexibility of the RdRp. The ΔΔSVib ENCoM value of A97A RdRp mutant in comparison to the wild RdRp is 1.228 kcal.mol-1.K-1, while that of P323I is -0.508 kcal.mol-1.K-1. Both these mutants were estimated to decrease RdRp flexibility. On the other hand, the Y606C ΔΔSVib ENCoM value is 0.844 kcal.mol^−1^.K^−1,^ which is an indication of increased RdRp flexibility. The vibrational entropy energy difference between the wild-type RdRp and mutated RdRp is presented in Figure 1. 

### 3.3. Mutations Cause Alteration in Secondary Structure of Proteins

**Figure 2:**
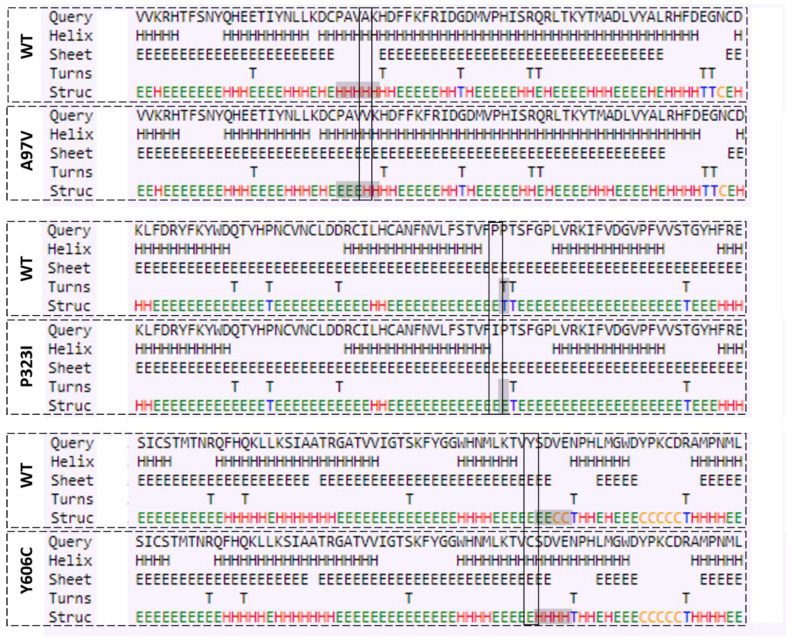
Prediction of secondary structure of RdRp protein. The difference in secondary structure between Wuhan (WT) and Saudi isolates are highlighted with position of dashed box in respective panels.

In order to evaluate the mutations’ effect on RdRp secondary structure elements, secondary structure analysis was carried out. Comparative analysis of secondary structure elements between the wild RdRp and mutants can be depicted in Figure 2. All the identified mutations in general do not affect the secondary structure elements of the position of the wild type. However, the A97V mutations were found to alter the five-helix string (present in the wild) and shorten it to the three-helix string upon mutation. P232I seems to have less impact on the overall structure element, whereas Y606C was revealed to produce a three-helix string right after the single mutation position in comparison to the wild type. The secondary structure analysis indicates that the mutation might affect the structure elements’ composition and patterning, which in turn might effect the RdRp structure and function, but this needs to be validated and studied further. 

### 3.4. Intramolecular Interactions Are Altered Due to Mutations in Proteins

**Figure 3:**
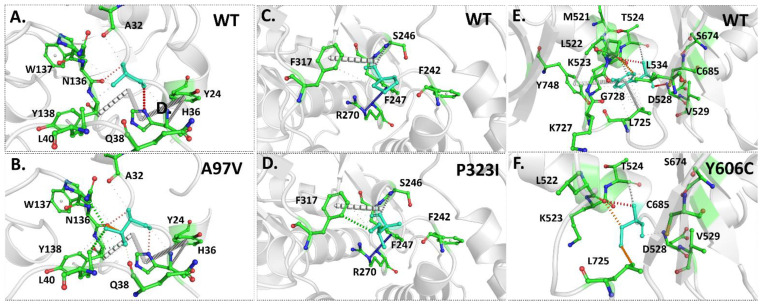
Effect of amino acid substitution on interatomic interactions of RdRp. (**A**,**B**) show the interatomic interactions mediated by wild type and A97V. (**C**,**D**) show the interatomic interactions mediated by wild type and P323I. (**E**,**F**) show the interatomic interactions mediated by wild type and Y606C. Wild-type and mutant residues are colored in cyan and are also represented as sticks alongside the surrounding residues (green), which are involved in any type of interactions.

A mutation within the amino acid sequence may bring about a change in the protein’s structure and function [43]. Therefore, the alteration of interatomic intramolecular interactions caused by identified mutations within the RdRp enzyme was assessed using DynaMut webserver. The substitution of the hydrophobic residue alanine (A) with hydrophobic valine (V) changes the interaction pattern, leading to the formation of two hydrogen bonds between mutant V97 and N136 and L40 (Figure 3A,B). These changes can provide more stability and less flexibility to the protein, which supports previous experiments. Likewise, mutation of P with hydrophobic I residue at position 323 induces the formation of hydrogen bonds with F317, which may result in the protein stability increasing (Figure 3C,D). However, a change from small hydrophobic V to C residue at position 606 seems to disrupt several intramolecular interactions, causing a destabilization effect (Figure 3E,F). These results were inconsistent with the above obtained results. 

### 3.5. Effect of Mutations on Protein Structural Conformation and Dynamic Stability

**Figure 4:**
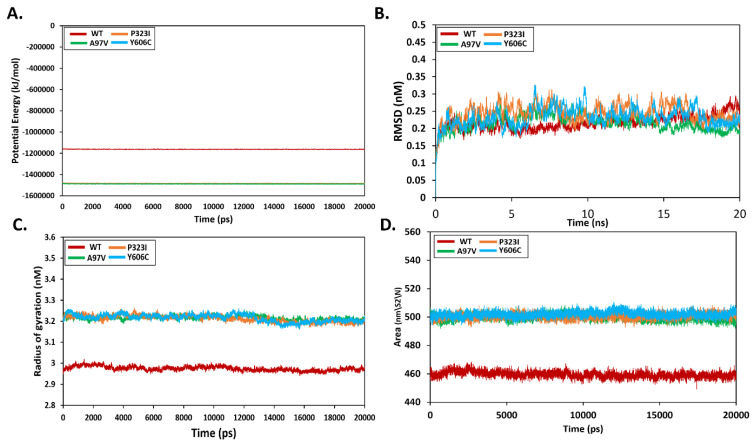
Molecular dynamics simulation analysis of wild-type and mutant RdRp enzymes. (**A**)—potential energy, (**B**)—RMSD, (**C**)—gyration, (**D**)—SASA.

To understand the dynamic stability of the apo wild-type and mutant RdRp enzymes, molecular dynamics simulations were carried out for length of 20 ns. The potential energy of the systems was determined first, which revealed that the mutant is in contrast to the wild type. The mutant RdRp was revealed to be more relaxed and unstrained compared to the wild-type RdRp (Figure 4A). The carbon alpha root-mean-square deviation (CαRMSD) was measured first for all systems (Figure 4B). In general, the wild as well as the mutants were seen in relative stable equilibrium. The wild RdRp in particular was observed in low dynamics across the length of simulation time, except towards the end where the enzyme achieved a slightly higher dynamic compared to the mutant enzymes. The A97V among the mutants was the most stable in the simulated time, and a good overall stability was noticed. These results were validated by calculating the radius of gyration (RoG) for the systems (Figure 4C). The RoG findings are mostly in line with the CαRMSD and demonstrate that the wild RdRp has a much lower RoG pattern. This points to greater compactness of the enzyme and more stable behavior. On contrast, the mutants have higher RoG and are reported to have a highly relaxed structure. Further, to access the stability, the folding and accessibility of water to the protein were calculated by solvent-accessible surface area (SASA) analysis (Figure 4D). Lower SASA values demonstrate tight packing of the protein folding and less flexibility. This further defines less access of solvent molecules and other interacting partners to bind the enzyme. It can be interpreted that upon mutations, the RdRp showed more solvent accessibility and impacted the enzyme’s intermolecular interaction dynamics and folding, functionality and overall stability dynamics. 

### 3.6. Effect of Mutations on Binding and Stability of Remdesivir

**Figure 5:**
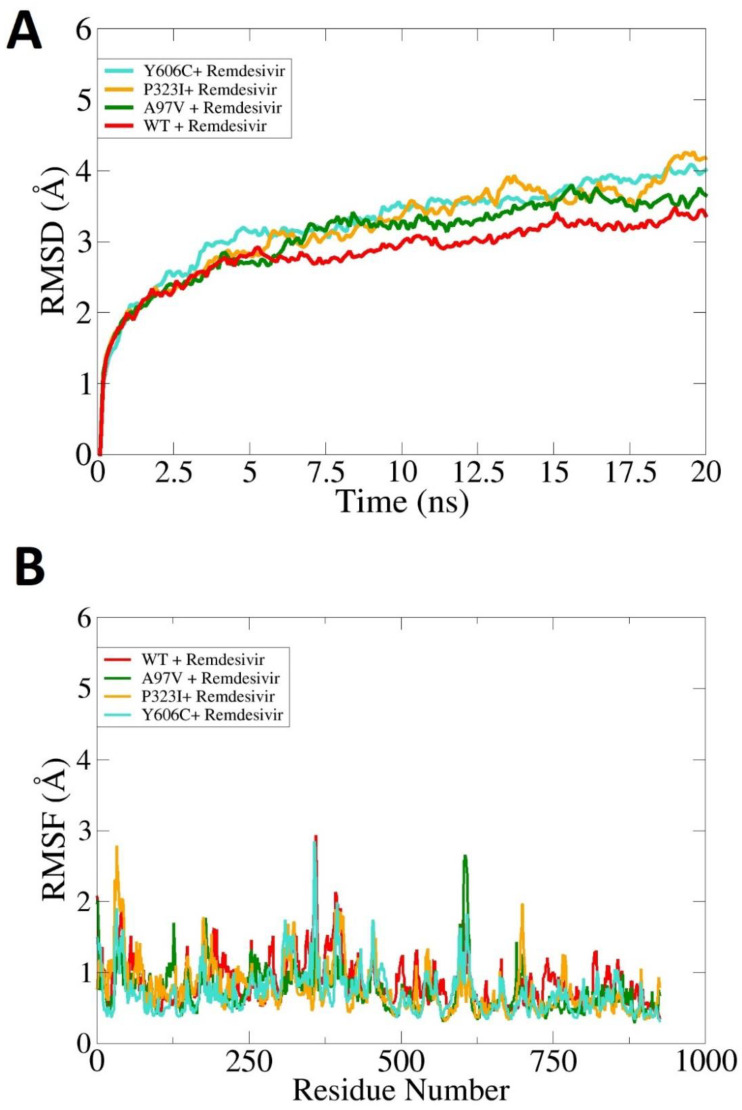
Molecular dynamics simulation analysis of WT-RdRp and mutants. (**A**)—RMSD and (**B**)—RMSF.

The effect of mutations on remdesivir with RdRp was elucidated by docking the remdesivir with both the wild type and mutants. It has been reported that the CαRMSD of the WT-RdRp complex with remdesivir is more stable compared to the rest of the mutants. The mutation in RdRp results in more flexibility being conferred to the enzyme’s flexible loops, which in turn make the enzyme more dynamic. The wild type, on the other hand, makes the enzyme structure more compact and allows for stable dynamics. The WT-RdRp and mutants’ RMSD are given in Figure 5A. The residue-wise flexibility of the WT-RdRp and mutants is depicted in Figure 5B, where it can be clearly observed that the WT-RdRp has more stable residue fluctuations compared to the rest of the mutants. 

Next, the molecular dynamics simulation trajectories were used for the calculation of binding free energies for the wild type and mutants with remdesivir using the MMGBSA method. The net binding free energies of the WT-RdRp complex with remdesivir, A97V-RdRp complex with remdesivir, P232I-RdRp complex with remdesivir and Y606C-RdRp complex with remdesivir are −50.76 kcal/mol, −44.96 kcal/mol, −47.2 kcal/mol and −41.34 kcal/mol, respectively. The wild-type system, as indicated by molecular dynamics analysis, was found the most stable in terms of intermolecular interaction and docked conformation, followed by P232I, A97V and Y606C. In all the systems, van der Waals energy was found dominant, and also the electrostatic energy plays a significant role in overall stability. Complete details of binding energies are tabulated in Table 4.

## 4. Conclusions

Due to the potential impact of some of these mutations on the specificity of diagnostic tests and vaccines and therapeutic development aimed at these sites, it is crucial to closely monitor the emergence of mutations throughout the duration of this ongoing pandemic. High numbers of mutations in the SARS-CoV-2 genome allow for efficient survival of the virus, allowing the virus to fit itself to the changing host immune responses, which in turn make it difficult to effectively manage the virus. Therefore, it is vital to understand the virus mutations capable of evading the virus and escaping from being therapeutically managed. In this study, three mutations—A97V, P323I and Y606C—were identified in different RdRp enzyme sequences extracted from Saudi Arabia isolates when aligned with reference Wuhan SARS-CoV-2 RdRp. The mutations were identified to affect the enzyme structure dynamics and stability and to alter interactions with the remdesivir ligand. Hence, studies are needed to explore the mutations’ impact on the RdRp structure–function activity and use that knowledge in further studies to design better therapeutics. 

## Figures and Tables

**Table 1:** Details of SARS-CoV-2 ORF1ab polyprotein sequences used in the analysis.

S. No.	Accession Number
1	YP_009724389
2	QMS50985
3	QMS50997
4	QMS51009
5	QMS51021
6	QMS51033
7	QMS51045
8	QMS51057
9	QMS51069
10	QMS51081
11	QMS51093
12	QMS51105
13	QMS51117
14	QMS51129
15	QMS51141
16	QMS51153
17	QMS51165
18	QMS51177
19	QMS51189
20	QMS51201
21	QMS51213
22	QMS51225
23	QMS51237
24	QMS51249
25	QMS51261
26	QMS51273
27	QMS51285
28	QMS51297
29	QMS51309
30	QMS51321
31	QLH56060
32	QLH56072
33	QLH56084
34	QLH56096
35	QLH56108
36	QLH56120
37	QLH56132
38	QLH56144
39	QLH56156
40	QLH56168
41	QLH56180
42	QLH56192
43	QLH56204
44	QLH56216
45	QLH56228
46	QLH56240
47	QLH56252
48	QKU37019
49	QKU37031
50	QKU37043
51	QKU37055
52	QKU37067
53	QKU37079
54	QKU37091
55	QKU37103
56	QKU37115
57	QKU37127
58	QKU37139
59	QKU37151

**Table 2:** Details of identified mutations.

Mutation ID	Wild-Type Residue	Position of Mutation	Mutated Residue	Frequency
A97V	A	97	V	1
P323I	P	323	I	56
Y606C	Y	606	C	1

**Table 3:** The values of change in ΔΔS ENCoM and ΔΔG (kcal/mol) due to the mutations in RdRp.

S. No	Wuhan Isolate	Saudi Isolate	AA Position	ΔΔS ENCoM	ΔΔG DynaMut	ΔΔG mCSM	ΔΔG SDM	ΔΔG DUET	Effect
1	A	V	97	4.117	1.397	−0.271	−1.270	−0.242	Stabilizing
2	P	I	323	0.406	1.017	−0.251	1.500	0.454	Stabilizing
3	Y	C	606	−0.984	−0.675	−1.675	−1.200	−1.721	Destabilizing

**Table 4:** Molecular dynamics simulation trajectory-based binding free energies in kcal/mol for wild type and mutants with remdesivir.

Energy Parameter	WT-RdRp Complex with Remdesivir	A97V-RdRp Complex with Remdesivir	P232I -RdRp Complex with Remdesivir	Y606C-RdRp Complex with Remdesivir
**VDWAALS**	−40.68	−39.33	−41.49	−37.51
**EEL**	−25.28	−22.00	−23.22	−19.67
**Delta G gas**	−65.96	−61.33	−64.71	−57.18
**Delta G solv**	15.20	16.37	17.51	15.84
**Delta Total**	−50.76	−44.96	−47.2	−41.34

## Data Availability

The data presented in this study are available within the article.
